# Proline Dehydrogenase/Proline Oxidase (PRODH/POX) Is Involved in the Mechanism of Metformin-Induced Apoptosis in C32 Melanoma Cell Line

**DOI:** 10.3390/ijms23042354

**Published:** 2022-02-21

**Authors:** Ilona Oscilowska, Karol Rolkowski, Weronika Baszanowska, Thi Yen Ly Huynh, Sylwia Lewoniewska, Magdalena Nizioł, Magdalena Sawicka, Katarzyna Bielawska, Paweł Szoka, Wojciech Miltyk, Jerzy Palka

**Affiliations:** 1 Department of Analysis and Bioanalysis of Medicines, Medical University of Bialystok, Kilinskiego 1, 15-089 Bialystok, Poland; ilona.zareba@gmail.com (I.O.); magdalena.niziol@umb.edu.pl (M.N.); sawicka.magdalenamaria@gmail.com (M.S.); katarzyna.bielawska@umb.edu.pl (K.B.); pawelszoka@wp.pl (P.S.); wojciech.miltyk@umb.edu.pl (W.M.); 2 Department of Medicinal Chemistry, Medical University of Bialystok, Kilinskiego 1, 15-089 Bialystok, Poland; karol.rolkowski@gmail.com (K.R.); w.baszanowska22@wp.pl (W.B.); htyly79@gmail.com (T.Y.L.H.); sylwialewoniewska123@wp.pl (S.L.)

**Keywords:** PRODH/POX, proline dehydrogenase, proline oxidase, metformin, AMPK, melanoma

## Abstract

The role of proline dehydrogenase/proline oxidase (PRODH/POX) in the mechanism of antineoplastic activity of metformin (MET) was studied in C32 melanoma cells. PRODH/POX is a mitochondrial enzyme-degrading proline that is implicated in the regulation of cancer cell survival/apoptosis. The enzyme is activated by AMP kinase (AMPK). It has been found that MET induced a significant decrease in cell viability and DNA biosynthesis accompanied by an increase in the expressions of AMPK and PRODH/POX in C32 cells. The mechanism for MET-dependent cytotoxicity on C32 cells was found at the level of PRODH/POX-induced ROS generation and activation of Caspase-3 and Caspase-9 expressions in these cells. The effects were not observed in MET-treated PRODH/POX knock-out C32 cells. Of interest is an MET-dependent increase in the concentration of proline, which is a substrate for PRODH/POX. This phenomenon is due to the MET-dependent inhibition of collagen biosynthesis, which is the main proline-utilizing process. It has been found that the underlying mechanism of anticancer activity of MET involves the activation of AMPK, PRODH/POX, increase in the cytoplasmic concentration of proline, inhibition of collagen biosynthesis, and stimulation of PRODH/POX-dependent ROS generation, which initiate the apoptosis of melanoma cells.

## 1. Introduction

Malignant melanoma is mostly a skin cancer that is derived from melanocytes [1,2]. According to epidemiological data, the number of newly diagnosed cases has increased more than threefold in the last 30 years [1,2]. The etiopathogenesis of cutaneous melanoma is not fully understood. It involves many genetic, metabolic, and environmental factors. In most cases of skin cancer, surgical methods are ineffective; therefore, pharmacological methods of treatment of the disease are considered [3,4].

Up to date, metformin (MET) is the first-line drug in the pharmacotherapy of type II diabetes. It inhibits intestinal glucose absorption, gluconeogenesis, and stimulates glycolysis and tissue sensitivity to insulin, contributing to hypoglycemia [5]. Studies of the last decade indicate that MET could be considered as an approach to the pharmacotherapy of melanoma. It has been well documented that MET inhibits melanoma cell growth both in vitro and in vivo [6,7,8,9], which has been proved in several clinical trials [10,11]. 

However, the link between MET-induced glycolysis and its anticancer potential is poorly understood. One of the effects of metformin is the activation of AMP kinase (AMPK) [7,12,13,14,15] and inhibition of mitochondrial respiration [5].

AMPK is activated by phosphorylation when the AMP/ATP ratio rises. This process stimulates oxidative phosphorylation to restore normal ATP levels and inhibits energy expenditure, such as cell proliferation [16,17]. Therefore, AMPK is regulated especially in conditions of energy shortage (e.g., starvation) and hypoxia [17] to inhibit anabolic processes and stimulate catabolism. One of the energy substrates in cancer cells is proline, which is derived from protein degradation, mainly collagen. Proline is degraded in mitochondria by proline dehydrogenase/proline oxidase (PRODH/POX) [18,19]. The enzyme is upregulated by AMPK [20]. 

PRODH/POX catalyzes the conversion of proline into ∆1-pyrroline-5-carboxylate (P5C). During this process, electrons are transported to the electron transport chain, producing ATP for survival [18,21,22], or they directly reduce oxygen, producing reactive oxygen species (ROS) for apoptosis/autophagy [23,24,25,26]. Therefore, PRODH/POX may play a dual role, but the mechanism that switches PRODH/POX from cancer growth-inhibiting to growth-supporting factor is unknown. PRODH/POX-dependent inhibition of cancer cell proliferation may result from ROS signaling [24,27,28]. The enzyme is upregulated by p53. Transcriptional regulation of PRODH/POX by p53 was found in the PRODH/POX promoter, containing a p53-response element [29,30,31]. 

Another factor that determines PRODH/POX-dependent functions is proline availability for the enzyme. Prolidase [E.C.3.4.13.9], the cytoplasmic enzyme releasing proline from imidodipeptides, is an important regulator of free proline in the cytoplasm [32,33,34,35,36]. However, it seems that the most important role in the regulation of proline concentration in the cytoplasm is collagen biosynthesis [37], which in this context may function as a sink for free proline. 

The aim of the study is the identification the mechanism of anticancer activity of MET. 

## 2. Results

The hypothesis was provided that metformin (MET) evokes pro-apoptotic potential by inducing PRODH/POX-dependent apoptosis in melanoma cells. The studies were performed on melanoma C32 cells, expressing PRODH/POX and PRODH/POX knock-out C32 cells (C32^POX−^). 

**Figure 1:**
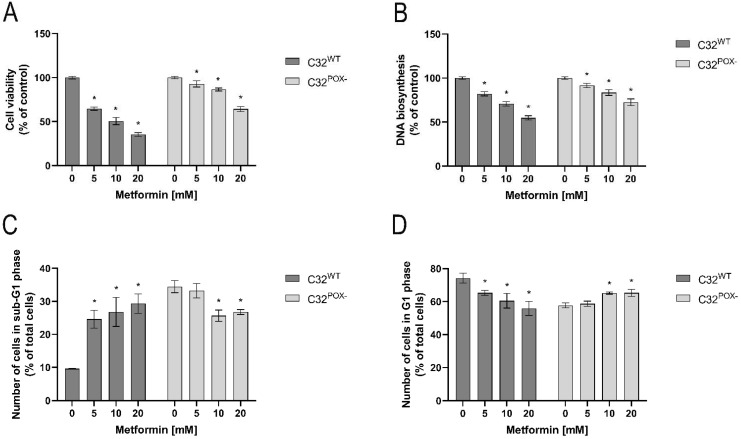
Cell viability (**A**), DNA biosynthesis (**B**), analysis of sub-G1 (**C**) and G1 (**D**) phases of cell cycle in C32 and C32^POX−^ cells upon treatment with metformin (0–20 mM, 24 h). The mean values with standard deviation (SD) from 3 experiments performed in duplicates are presented. Asterisks * indicate statistical differences between studied cells compared to controls at *p* < 0.001.

As shown in Figure 1, MET inhibited cell viability (A) and DNA biosynthesis (B) in both cell lines in a concentration-dependent manner. However, the inhibition was more pronounced in C32 cells than in C32^POX−^. At 20 mM, MET decreased C32 cell viability to about 40% and C32^POX−^ to about 70% of control. DNA biosynthesis was similarly affected by MET treatment in both cell lines; however, it was to a lower extent. Moreover, as presented in Figure 1C,D, in C32 cells, MET increased in a dose-dependent manner the number of cells in the sub-G1 phase (characteristic for apoptotic cells) and decreased the number of cells in the G1 phase (characteristic for normal cells). In C32^POX−^, an opposite effect of MET was found. The data suggest that MET cytotoxicity could undergo apoptosis in C32 cells.

**Figure 2:**
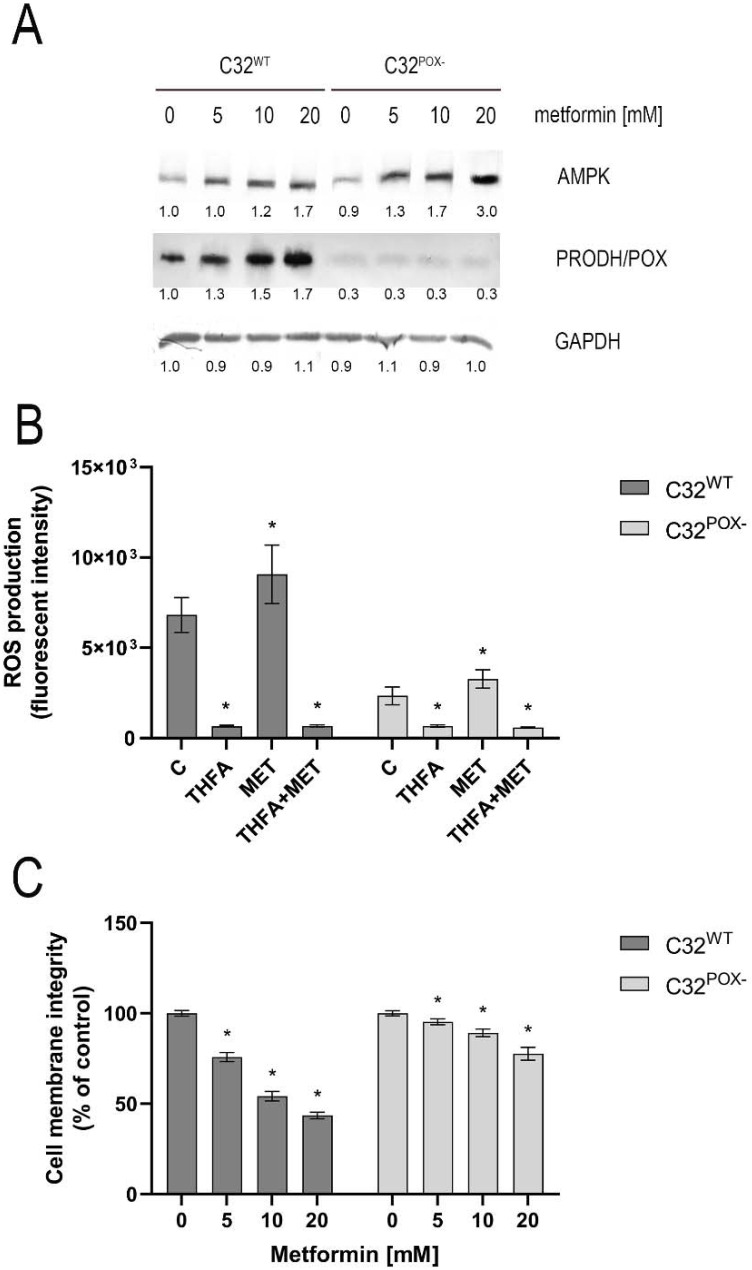
AMPK and PRODH/POX expression (**A**), ROS formation (**B**), and cell membrane integration (**C**) in C32 and C32^POX−^ cells treated with metformin (0–20 mM, MET) for 24 h. THFA (PRODH/POX inhibitor) was used at a concentration of 200 µM. Representative Western blot images were shown (the mean values of densitometric analysis after GAPDH normalization as a ratio versus control were presented below each blot). Supplementary Materials contain statistical analysis of the evaluated proteins (Supplementary Material, Figures S1 and S2). The mean values with standard deviation (SD) from 3 experiments performed in duplicates are presented. Asterisks * indicate statistical differences between studied cells compared to controls at *p* < 0.001. C—control, THFA—tetrahydrofuroic acid, MET—metformin.

Since MET is known to induce AMPK [6,15] and this kinase upregulates PRODH/POX [21], the expression of these proteins was measured in MET-treated cells by Western immunoblotting. As shown in Figure 2A, MET in a dose-dependent manner strongly stimulated AMPK and PRODH/POX expressions in melanoma C32 cells. In MET-treated C32^POX−^, AMPK expression was much higher than in C32 cells and PRODH/POX expression was not detected. An increase in PRODH/POX expression in MET-treated C32 cells was accompanied by an increase in ROS formation (Figure 2B) and a decrease in cell membrane integrity (Figure 2C). These effects were also seen in C32^POX−^; however, it was to a much lower extent. 

An increase in ROS generation in MET-treated cells was counteracted by THFA, which is a specific PRODH/POX inhibitor [38,39].

**Figure 3:**
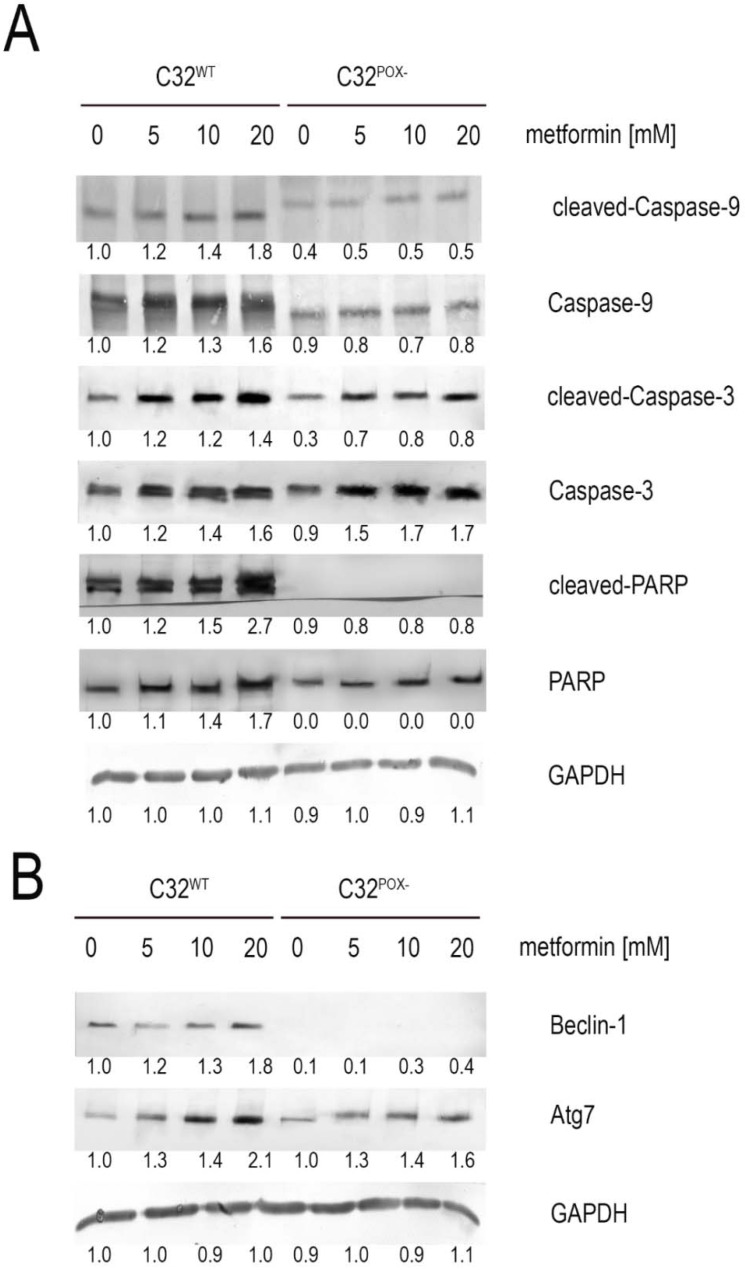
Expression of (**A**) apoptosis markers (cleaved-Caspase-9, Caspase-9, cleaved-Caspase-3, Caspase-3, cleaved-PARP, and PARP) and (**B**) autophagy markers (Beclin-1 and Atg7). Representative Western blot images are shown (the mean values of densitometric analysis after GAPDH normalization as a ratio versus control were presented below each blot). Supplementary Materials contain statistical analysis of the evaluated proteins (Supplementary Material, Figures S3–S8).

Functional significance of MET-dependent cytotoxicity and ROS formation was found at the level of expression of apoptosis (Caspase-3, Caspase-9, PARP) and autophagy (Beclin-1, Atg7) markers. As shown in Figure 3A, active Caspases-3, -9, and PARP were strongly expressed in MET-treated C32^WT^ cells and slightly expressed in MET-treated C32^POX−^. MET also stimulated Beclin-1 and Atg7 expressions in C32^WT^ cells, while in C32^POX−^, Beclin-1 was not detected in contrary to Atg7, which was also stimulated by MET (Figure 3B). It suggests that PRODH/POX-dependent ROS formation contributes to apoptosis and the autophagic death of C32^WT^ cells.

**Figure 4:**
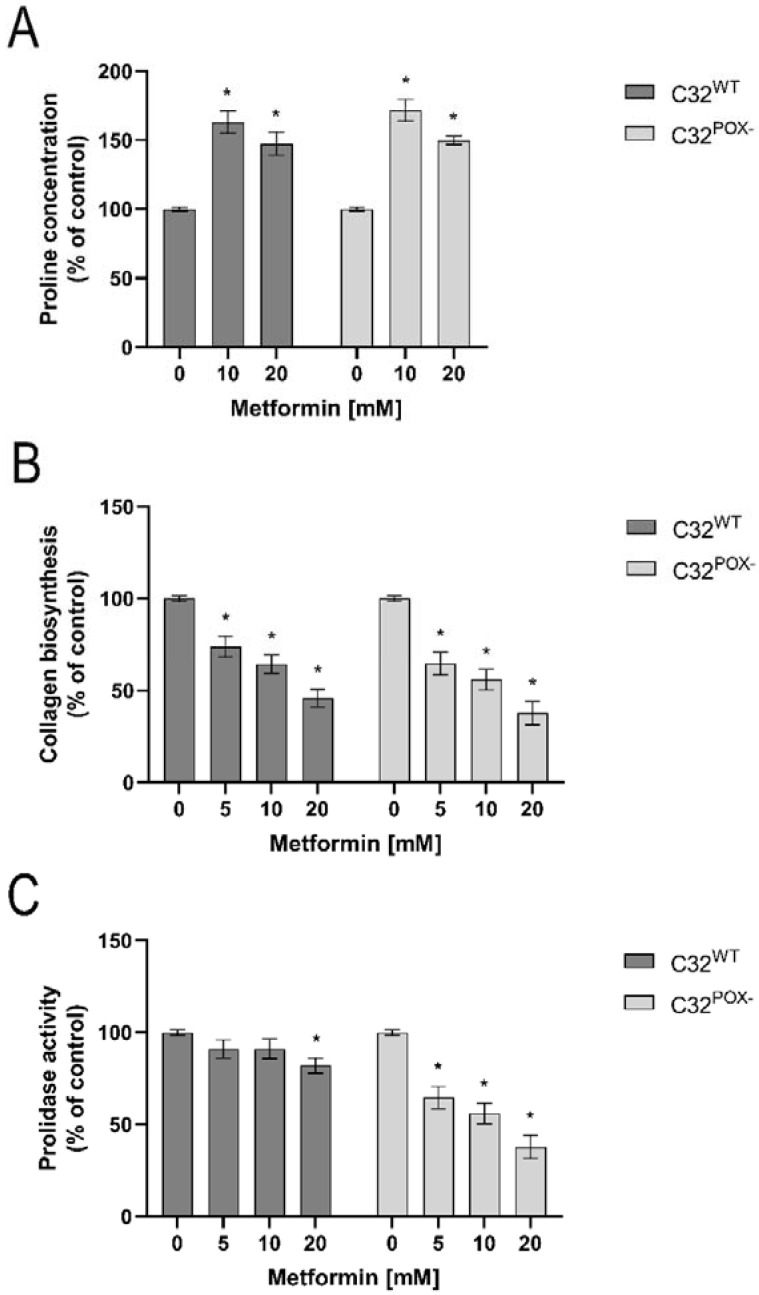
Proline concentration (**A**), collagen biosynthesis (**B**), and prolidase activity (**C**) in C32^WT^ and C32^POX−^ cells upon treatment with metformin (0–20 mM) for 24 h. The mean values with standard deviation (SD) from 3 experiments performed in duplicates are presented. Asterisks * indicate statistical differences between studied cells compared to controls at *p* < 0.001.

PRODH/POX-induced apoptosis is dependent on substrate availability for the enzyme. Since proline availability for PRODH/POX is regulated by prolidase (proline supporting enzyme) and collagen biosynthesis (proline utilizing process), the effect of MET on the processes was studied. As shown in Figure 4, MET similarly increased proline concentration (Figure 4A) and inhibited collagen biosynthesis (Figure 4B) in both cell lines. However, prolidase activity was inhibited by MET (in a dose-dependent manner) in C32^POX−^, while in C32^WT^ cells, the enzyme was significantly affected only at 20 mM of MET. Nevertheless, this property of metformin did not reduce the concentration of free proline in the cells. The data suggest that MET supports proline for PRODH/POX-dependent functions by inhibiting collagen biosynthesis.

## 3. Discussion

Several lines of evidence presented in this paper suggest that the mechanism of the anticancer activity of MET involves PRODH/POX-dependent ROS generation in melanoma cells. We suggest that important players in this process are AMPK, as an inducer of PRODH/POX, and collagen biosynthesis, as a regulator of proline availability for the enzyme. MET was found to (i) induce AMPK-stimulating PRODH/POX and (ii) inhibit collagen biosynthesis, facilitating proline availability for PRODH/POX-dependent ROS generation that induces apoptosis in melanoma cells. 

It seems that MET induces the reprogramming of energetic metabolism in melanoma cells. The melanoma cells, as well as many other cancer cells, are characterized by enhanced aerobic glycolysis yielding lactate, which is known as a Warburg effect [40,41]. It ensures the rapid production of ATP from glucose to support cancer cell proliferation [42,43]. The conversion of pyruvate into lactate ensures a high NAD+/NADH ratio that accelerates glycolysis but deregulates redox potential [44]. The process of ATP production from glucose by the Warburg effect is less efficient than during mitochondrial oxidative phosphorylation. Therefore, cancer cells need another source of energy. Particularly, proline could serve as an alternative source of energy. A large quantity of proline comes from protein degradation, mostly from the most abundant extracellular protein—collagen. Warburg’s effect facilitates protein degradation as an alternative source of energy. Moreover, the Warburg effect contributes to the augmentation of glutaminolysis, leading to an increase in proline concentration and its metabolites [45]. Proline, ornithine, and glutamate are interconvertible amino acids with an intermediate of P5C, linking TCA and urea cycles with glutamine metabolism. Therefore, proline could be an energetic substrate in a reaction catalyzed by PRODH/POX. However, in cancer cells, proline metabolism by PRODH/POX is limited, since lactate inhibits its activity [46]. In such conditions, energy shortage activates AMPK; however, it is not effective in the stimulation of PRODH/POX [47,48] because of high lactate concentration that inhibits PRODH/POX [46]. 

One of the well-recognized effects of MET is inhibition of respiratory complex I [6]. It suggests that electrons from PRODH/POX-dependent proline degradation are directly accepted by oxygen, generating ROS-induced apoptosis. Since MET upregulates PRODH/POX, as was shown in this report, the process is agitated in MET-treated melanoma cells. Whether this is the universal mechanism in cancer cells needs to be explored. However, several lines of evidence support such a mechanism of MET-dependent apoptosis. 

Several studies showed that the proline concentration is increased in various cancers [49,50]. It seems that the increase in proline level is due to the degradation of extracellular matrix collagen type I, which is the protein containing a high amount of proline [51]. Energy shortage induces matrix metallopreoteinases (MMP)-2 and -9 [20], suggesting the mechanism for an increase in cellular proline concentration. In such a case, cancer cells may select proline as an alternative energy source. In vivo proline has an advantage over fatty acids and glutamine, which similar to glucose require delivery by circulation. Therefore, proline may represent an energy sense molecule and energy substrate. However, a critical factor that mediates adaptation for such metabolic change is AMPK [52,53]. This kinase inhibits energy-consuming processes and activates energy-producing processes to restore energy homeostasis during stress situations [54,55]. Proline could be also generated from glutamine/glutamate as well as from ornithine [56], linking tricarboxylic acid (TCA) and Urea cycles with proline metabolism. 

In this report, evidence was provided that MET induced the expression of AMPK, PRODH/POX, active Caspase 3, -9, and PARP in a dose-dependent manner. The effect was not found in C32^POX−^ cells deprived of PRODH/POX. It suggests that PRODH/POX represents an underlying mechanism for the initiation of apoptosis in MET-treated melanoma cells. Interestingly, some autophagy markers (Beclin-1, Atg7) were also upregulated in MET-treated melanoma cells. PRODH/POX was found to induce autophagy in some micro-environmental conditions in breast cancer cells [57,58,59]. It cannot be excluded that both processes run in parallel in MET-treated cells, and the result is determined by the complex of metabolic processes in the melanoma cells. Probably the most important is energetic metabolism, particularly involving glucose metabolism. Recently, it has been found that MET inhibited glycolysis as demonstrated by a drastic increase in intracellular glucose content and dependently on the presence or absence of glutamine, the drug affected metabolites of TCA and urea cycles in breast cancer MCF-7 cells [60]. It suggests that MET-induced glucose starvation contributes to the acquisition of energy from other sources, e.g., proline. In fact, previously, we provided evidence that metformin induced apoptosis in both WT and PRODH/POX knock-out MCF-7 cells, however only when cultured in the absence of glutamine, while the presence of glutamine in cell culture medium facilitated the pro-survival phenotype of the cells [60]. Metabolomic analysis suggested that glycolysis is tightly linked to glutamine and proline metabolism in these cells, creating metabolic conditions for energy production and proline availability for PRODH/POX-dependent functions. Metformin treatment of both cell lines (WT and PRODH/POX knock out MCF-7 cells) cultured in glutamine free medium contributed to glucose starvation, facilitating the pro-apoptotic phenotype of these cells, as detected by increase in the expression of active Caspase-7 and PARP. Caspase-7 is known as an executioner protein of apoptosis activated by Caspase-8 (extrinsic pathway) and Caspase-9 (intrinsic pathway). These results provided insight into the potential mechanism of anticancer activity of metformin and suggested that PRODH/POX is an important player in the apoptosis. In further unpublished studies on PRODH/POX-dependent apoptosis in MCF-7 cells, we found that the stimulation of PRODH/POX (by PPAR-ϒ activation) induced reactive oxygen species (ROS) generation and ROS-dependent apoptosis (accompanied by an increase in the expression of active Caspase-9), while PRODH/POX knock-out abolished ROS-dependent apoptosis; however, it induced apoptosis by the extrinsic pathway (activation of Caspase-8). The interplay between PRODH/POX-dependent extrinsic and intrinsic pathways is probably metabolic context dependent. It seems that in conditions of PRODH/POX knock-out and inhibition of collagen biosynthesis (proline consuming process), proline concentration drastically increases activating extrinsic apoptosis. Whether this is the case is currently under investigation.

Interestingly, in present studies, we found that metformin induced PRODH/POX-dependent apoptosis in melanoma cells, which is characterized by an intense biosynthesis of collagen [61]. In this case, PRODH/POX knock-out did not induce apoptosis. It seems that in the melanoma cells with a high capacity to utilize proline for collagen biosynthesis, the proline concentration is not high enough to induce extrinsic apoptosis. However, the stimulation of PRODH/POX by metformin induced ROS-dependent apoptosis, while PRODH/POX knock-out abolished the effect. Therefore, we suggest that the role of PRODH/POX in the apoptosis/survival of cancer cells is not a zero-one system but rather depends on metabolic context of a specific cell type.

PRODH/POX cooperate with P5C reductase (P5CR) participating in proline turnover between mitochondria and cytoplasm. The conversion of proline to P5C that is shuttled between mitochondria and the cytosol is coupled to glucose metabolism by the pentose phosphate pathway [18,19,20,29]. It is vital in the maintenance of redox balance in a cell due to the participation of NAD+/NADH in the conversion of P5C to proline. Moreover, P5C is converted by P5C dehydrogenase (P5CDH) to glutamate, which is a precursor of α-ketoglutaric acid—a component of the TCA cycle. Such a cycle of proline/P5C between mitochondria and cytoplasm could be responsible for ROS generation and apoptosis induction. It may undergo in case of inhibition of proline utilization for collagen biosynthesis. Although the role of collagen biosynthesis in the mechanism for switch between ATP and ROS production in mitochondria is hypothetical, it is partially supported by data showing that 2-metoxyestradiol, an inhibitor of collagen biosynthesis [62], induces apoptosis and autophagy in adenocarcinoma cells [63]. The tendency was found in breast cancer tissue, which evoked enhanced prolidase activity and decreased collagen content [64]. Recently, metformin was shown to inhibit collagen biosynthesis in fibroblasts [65,66]. It seems that metformin-dependent inhibition of collagen biosynthesis and metformin-induced PRODH/POX expression accelerates the proline cycle, contributing to ROS generation and apoptosis in cancer cells. 

## 4. Materials and Methods

### 4.1. PRODH/POX Knock Uut CRISPR-cas9 DNA Plasmid Purification

The sgRNAs for PRODH/POX (CRISPR All-In-One Non-Viral Vector with spCas9) were ordered by ABM Company (Richmond, VA, Canada). The vector with expression construct (directed against PRODH1 isoform) was transformed into Escherichia coli DH5α and grown in Luria–Bertani (LB) media supplemented with 100 µg·mL^−1^ ampicillin at room temperature for 24 h, as described previously [60]. The targeted plasmid was extracted by a plasmid DNA purification kit (Nucleobond Xtra Midi/Maxi, MACHERY-NAREL GmbH, Düren, Germany). After being precipitated by isopropanol, the purified samples were washed by 70% ethanol solution and then followed by a DNA cleaning-up step by a GeneMATRIX Basic DNA Purification Kit (EURX, E3545-01 protocol 1, Gdansk, Poland). The purified DNA concentration was estimated by NanoDrop™ 2000/2000c Spectrophotometers (Thermo Fisher Scientific, Waltham, MA, USA).

### 4.2. Transfection into Melanoma Cell Line

C32 melanoma cells (catalog number: CRL-1585, purchased from ATCC) were cultured in the complete growth medium, DMEM 1X (Gibco, Carlsbad, CA, USA) containing 4.5 gL^−1^ glucose, L-glutamine, and pyruvate supplemented with 10% Fetal Bovine Serum (FBS) qualified (Gibco, Carlsbad, CA, USA), 1% penicillin/streptomycin (Invivogen, San Diego, CA, USA) at 37 °C in 5% CO_2_. Then, the cells were seeded into 6-well plates to reach 70–90% confluency. The amount of plasmid in the experiment was tested from 1 to 2 µg per well. Lipofectamine 2000 (Invitrogen, Thermo Fisher Scientific, Waltham, MA, USA) was used as a transfection reagent.

Prior to transfection, the plasmid was diluted with 50 µL of medium A, DMEM 1X (Gibco, Carlsbad, CA, USA) containing 4.5 gL^−1^ glucose, L-glutamine, and pyruvate supplemented with 1% penicillin/streptomycin (Invivogen, San Diego, CA, USA). 

The transfection solution containing 805.4 µL of medium A and 194.6 µL of lipofectamine reagent were gently mixed and then incubated at room temperature for 5 min before aliquoting 60 µL of the solution into a vial containing the diluted plasmid solution. The mixture of diluted plasmid and transfection solution was mixed gently and then incubated at room temperature for 20 min. 

The testing cells were washed by PBS 1X (sterile phosphate-buffered saline 1X, Gibco, Carlsbad, CA, USA) and freshly added with 1 mL of medium A. After 20 min incubation, the mixture of plasmid and transfection reagent was slowly added to cells and then incubated at 37 °C in 5% CO_2_ overnight. The following day, the transfected cells were selected in the complete growth medium with 1 µg·mL^−1^ of puromycin (Sigma-Aldrich, Saint Louis, MI, USA) in the same culture conditions for 10 days. The expression of PRODH/POX in transfected cells was checked by Western immunoblotting. Based on the results of expression level between wild-type C32 cells and transfected C32 cells, the PRODH/POX knock-out C32 cell line was selected for further stable clone generation. The process of the stable clone generation was manipulated with a serial dilution of the selected cells in the culture media to obtain 0.7 cell per well in a 96-well plate. The screening steps were completed with a random selection of cell clones. The PRODH/POX silencing in cell clones was checked by Western immunoblotting using an anti-PRODH/POX antibody (Santa Cruz, Dallas, TX, USA). The level of PRODH/POX knockdown is presented in (Supplementary Material Figure S1). The PRODH/POX knock-out C32 cells defined as C32^POX−^ cells were frozen in liquid nitrogen vapor and banked for further experiments.

### 4.3. Cell Culture and Treatment 

Melanoma (C32) cell lines were purchased from ATCC (ATCC, Manassas, VA, USA). The cells were grown in a DMEM cell culture medium (PanBiotech, Aidenbach, Germany) containing 10% fetal bovine serum (Gibco, Carlsbad, CA, USA) and 1% penicillin/streptomycin (Gibco, Carlsbad, CA, USA) at 37 °C in a humidified atmosphere of 5% CO_2_. For experiments, cells (4–7th passages) were treated with metformin at concentrations of 0–20 mM for 24 h. 

### 4.4. Cell Viability 

The cell viability of metformin-treated cells was measured using the MTT assay, as described previously [67]. Cell survival was calculated as a percentage of living cells when compared to control (0 mM of metformin, 100% survival). 

### 4.5. DNA Biosynthesis Assay 

DNA biosynthesis of C32^WT^ and C32^POX−^ cells was evaluated with the CyQUANT^®^ Cell Proliferation Assay (Thermo Fisher Scientific, Waltham, MA, USA) according to the previously described procedure [68].

### 4.6. Cell Cycle Analysis 

The cell cycle of C32^WT^ and C32^POX−^ cells upon metformin treatment (0–20 mM) for 24 h was analyzed as previously described [69]. 

### 4.7. ROS Generation Assessment 

Intracellular reactive oxygen species accumulation was measured using DCFH-DA as a fluorescent probe. Briefly, cells were pre-incubated with DCFH-DA (20 µM) in culture medium for 30 min, washed twice with PBS, and treated with increasing concentrations of metformin (MET, 0–20 mM) for 24 h, 200 µM tetrahydrofuroic acid (THFA, Sigma Aldrich, Saint Louis, MI, USA) or both compounds in DMEM. THFA (proline analogue) was used as a PRODH/POX inhibitor [70,71]. The fluorescent intensity was measured at an excitation/emission wavelength of 488/535 nm using TECAN Infinite^®^ M200 PRO (Männedorf, Switzerland). The results were presented as a fluorescence intensity using arbitrary units. 

### 4.8. Cell Membrane Integrity Assay 

Cell membrane integrity of metformin-treated cells was evaluated by using neutral red uptake (NRU) assay, which was described previously [68]. Cytotoxicity was calculated as a percentage of the control (0 mM of metformin, 100% of intact membranes). 

### 4.9. Preparation of Cell Lysates 

Cells were cultured in FBS-free DMEM with metformin (0–20 mM) for 24 h. Then, cells were collected as previously described [72]. Protein concentration was measured using the Lowry method [73]. Cell lysates were subjected to Western immunoblotting and prolidase activity measurement. 

### 4.10. Western Immunoblotting 

The Western immunoblotting analyses were performed as described previously [57]. The membranes were incubated with primary antibodies (all from CST and in 1:1000 dilution; Cell Signaling, Danvers, MA, USA) overnight, including anti-AMPKα, anti-Atg7, anti-Beclin-1, anti-cleaved Caspase-3, anti-Caspase-3, anti-cleaved Caspase-9, anti-Caspase-9, anti-cleaved PARP, anti-PARP, anti-PRODH, and anti-GAPDH. The bands were visualized using 1-Step™ NBT/BCIP Substrate Solution (Thermo Fisher Scientific, Waltham, MA, USA), and their intensities were semi-quantitatively calculated with ImageJ software (https://imagej.nih.gov/ij/). Western immunoblotting analysis was performed at least in triplicate.

### 4.11. Determination of Prolidase Activity 

The activity of prolidase was determined as described previously [58]. Enzyme activity was reported as nanomoles of proline released from the synthetic substrate (glycyl-proline) during 1 min per milligram of supernatant protein of cell homogenate. 

### 4.12. Collagen Biosynthesis Assay 

Collagen biosynthesis in C32^WT^ and C32^POX−^ cells was measured as described previously [59]. Radiometric measurement was run on a Liquid Scintillation Analyzer Tri-Carb 2810 TR (PerkinElmer, Waltham, MA, USA). 

### 4.13. LC-MS Analysis of Proline Concentration 

Proline concentration in C32^WT^ and C32^POX−^ cells was measured with the use of the method published by Klupczynska et al. [74]. Samples were analyzed using Agilent 1260 Infinity HPLC system coupled to Agilent 6530 Q-TOF mass spectrometry detector with electrospray ionization as an ion source in positive ionization mode. Samples were injected onto an HILIC column (Luna HILIC, 2 × 100 mm, 3 µm, Phenomenex, Torrance, CA, USA). Methanol-extracted cell lysates were collected in triplicates and injected in duplicates. Total protein concentration was used for normalization and presented as percentage of control. 

### 4.14. Statistical Analysis 

All experiments were run at least in triplicates, and the experiments were repeated twice. Data represent a mean ± standard deviation (SD). For statistical analysis, one-way analysis of variance (ANOVA) with Dunnett’s correction and *t*-test using GraphPad Prism 5.01 (GraphPad Software, San Diego, CA, USA) were used. Results were considered statistically significant at *p* < 0.001 and denoted by asterisks *.

## 5. Conclusions

**Figure 5:**
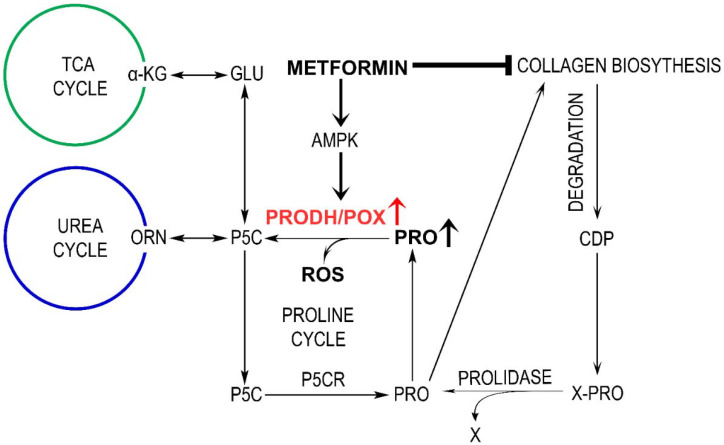
Mechanism of metformin-induced PRODH/POX-dependent apoptosis in melanoma C32 cells, linking TCA, urea cycles, and proline synthesis and degradation with collagen biosynthesis and degradation. ATP—adenosine triphosphate, CDP—collagen degradation products, PEPD—prolidase, PRO—proline, PRODH/POX—proline dehydrogenase/oxidase, PYCR—1-pyrroline-5-carboxylate reductase, P5C—1-pyrroline-5-carboxylate, ROS—reactive oxygen species, TCA—tricarboxylic acid cycle.

Collectively, our results indicate that the mechanism for MET-induced apoptosis in melanoma cells involves the upregulation of AMPK and PRODH/POX-dependent ROS formation and inhibition of collagen biosynthesis, increasing proline availability for PRODH/POX. The potential mechanism of this process is outlined in Figure 5.

## Data Availability

The datasets used and/or analyzed during the current study are available from the corresponding author on reasonable request.

## Supplementary Materials

The following supporting information can be downloaded at: https://www.mdpi.com/article/10.3390/ijms23042354/s1.

## Abbreviations

AMP	5′ adenosine monophosphate-activated protein
AMPK	AMP kinase
ATCC	American Type Culture Collection
Atg7	autophagy-related 7 protein
ATP	adenozyno-5′-trifosforan
C32	type of melanoma cells
CRISPR	clustered regularly interspaced short palindromic repeats
DCFH-DA	2′-7′dichlorofluorescin diacetate
DMEM	Dulbecco’s Modified Eagle Medium
DNA	deoxyribonucleic acid
FBS	fetal bovine serum
GAPDH	glyceraldehyde 3-phosphate dehydrogenase
HILIC	hydrophilic interaction liquid chromatography
HPLC	high-performance liquid chromatography
MCF-7	type of breast cancer cells
MET	metformin
MMP-2	metaloproteinase 2
MMP-9	metaloproteinase 9
MTT	3-(4,5-dimethylthiazol-2-yl)-2,5-diphenyltetrazolium bromide
NAD+	nicotinamide adenine dinucleotide
NADH	nicotinamide adenine dinucleotide hydrogen
NRU	neutral red uptake
p53	transcription factor p53
P5C	∆1-pyrroline-5-carboxylate
P5CR	P5C reductase
PARP	poly (ADP-ribose) polymerase
PBS	phosphate-buffered saline
POX	proline oxidase
PRODH	proline dehydrogenase
QTOF	quadrupole time-of-flight mass spectroscopy
ROS	reactive oxygen species
TCA	tricarboxylic acid cycle
THFA	tetrahydrofuroic acid

## References

[1] Wojciechowska U., Didkowska J., Michałek I., Olasek P., Ciuba A. (2018). Cancer in Poland in 2018. Polish National Cancer Registry.

[2] Rutkowski P., Wysocki P., Nowecki Z., Rudnicki L., Nasierowska-Guttmejer A., Fijuth J., Kalinka-Warzocha E., Świtaj T., Jeziorski A., Szacht M. (2015). Cutaneous melanoma—Diagnostic and therapeutic guidelines in 2016. Oncol. Clin. Pract..

[3] Rigel D.S., Friedman R.J., Kopf A.W., Polsky D. (2005). ABCDE—An evolving concept in the early detection of melanoma. Arch. Dermatol..

[4] Clark W.H., Elder D.E., Guerry D.t., Epstein M.N., Greene M.H., Van Horn M. (1984). A study of tumor progression: The precursor lesions of superficial spreading and nodular melanoma. Hum. Pathol..

[5] Rena G., Hardie D.G., Pearson E.R. (2017). The mechanisms of action of metformin. Diabetologia.

[6] Chaube B., Malvi P., Singh S.V., Mohammad N., Meena A.S., Bhat M.K. (2015). Targeting metabolic flexibility by simultaneously inhibiting respiratory complex I and lactate generation retards melanoma progression. Oncotarget.

[7] Janjetovic K., Harhaji-Trajkovic L., Misirkic-Marjanovic M., Vucicevic L., Stevanovic D., Zogovic N., Sumarac-Dumanovic M., Micic D., Trajkovic V. (2011). In vitro and in vivo anti-melanoma action of metformin. Eur. J. Pharmacol..

[8] Peppicelli S., Toti A., Giannoni E., Bianchini F., Margheri F., Del Rosso M., Calorini L. (2016). Metformin is also effective on lactic acidosis-exposed melanoma cells switched to oxidative phosphorylation. Cell Cycle.

[9] Jaune E., Rocchi S. (2018). Metformin: Focus on Melanoma. Front. Endocrinol..

[10] De Flora S., Ganchev G., Iltcheva M., La Maestra S., Micale R.T., Steele V.E., Balansky R. (2016). Pharmacological Modulation of Lung Carcinogenesis in Smokers: Preclinical and Clinical Evidence. Trends Pharmacol. Sci..

[11] Chae Y.K., Arya A., Malecek M.K., Shin D.S., Carneiro B., Chandra S., Kaplan J., Kalyan A., Altman J.K., Platanias L. (2016). Repurposing metformin for cancer treatment: Current clinical studies. Oncotarget.

[12] Han D., Li S.J., Zhu Y.T., Liu L., Li M.X. (2013). LKB1/AMPK/mTOR signaling pathway in non-small-cell lung cancer. Asian Pac. J. Cancer Prev..

[13] Salani B., Maffioli S., Hamoudane M., Parodi A., Ravera S., Passalacqua M., Alama A., Nhiri M., Cordera R., Maggi D. (2012). Caveolin-1 is essential for metformin inhibitory effect on IGF1 action in non-small-cell lung cancer cells. FASEB J..

[14] Guo Q., Liu Z., Jiang L., Liu M., Ma J., Yang C., Han L., Nan K., Liang X. (2016). Metformin inhibits growth of human non-small cell lung cancer cells via liver kinase B-1-independent activation of adenosine monophosphate-activated protein kinase. Mol. Med. Rep..

[15] Wang J., Gao Q., Wang D., Wang Z., Hu C. (2015). Metformin inhibits growth of lung adenocarcinoma cells by inducing apoptosis via the mitochondria-mediated pathway. Oncol. Lett..

[16] Gwinn D.M., Shackelford D.B., Egan D.F., Mihaylova M.M., Mery A., Vasquez D.S., Turk B.E., Shaw R.J. (2008). AMPK phosphorylation of raptor mediates a metabolic checkpoint. Mol. Cell.

[17] Hardie D.G. (2003). Minireview: The AMP-activated protein kinase cascade: The key sensor of cellular energy status. Endocrinology.

[18] Liu W., Phang J.M. (2012). Proline dehydrogenase (oxidase), a mitochondrial tumor suppressor, and autophagy under the hypoxia microenvironment. Autophagy.

[19] Phang J.M., Liu W., Hancock C., Christian K.J. (2012). The proline regulatory axis and cancer. Front. Oncol..

[20] Pandhare J., Donald S.P., Cooper S.K., Phang J.M. (2009). Regulation and function of proline oxidase under nutrient stress. J. Cell Biochem..

[21] Phang J.M., Donald S.P., Pandhare J., Liu Y. (2008). The metabolism of proline, a stress substrate, modulates carcinogenic pathways. Amino Acids.

[22] Donald S.P., Sun X.Y., Hu C.A., Yu J., Mei J.M., Valle D., Phang J.M. (2001). Proline oxidase, encoded by p53-induced gene-6, catalyzes the generation of proline-dependent reactive oxygen species. Cancer Res..

[23] Hu C.A., Donald S.P., Yu J., Lin W.W., Liu Z., Steel G., Obie C., Valle D., Phang J.M. (2007). Overexpression of proline oxidase induces proline-dependent and mitochondria-mediated apoptosis. Mol. Cell. Biochem..

[24] Liu Y., Borchert G.L., Surazynski A., Hu C.A., Phang J.M. (2006). Proline oxidase activates both intrinsic and extrinsic pathways for apoptosis: The role of ROS/superoxides, NFAT and MEK/ERK signaling. Oncogene.

[25] Martindale J.L., Holbrook N.J. (2002). Cellular response to oxidative stress: Signaling for suicide and survival. J. Cell. Physiol..

[26] Raha S., Robinson B.H. (2001). Mitochondria, oxygen free radicals, and apoptosis. Am. J. Med. Genet..

[27] Liu Y., Borchert G.L., Surazynski A., Phang J.M. (2008). Proline oxidase, a p53-induced gene, targets COX-2/PGE2 signaling to induce apoptosis and inhibit tumor growth in colorectal cancers. Oncogene.

[28] Liu W., Tang F., Deng Y., Li X., Lan T., Zhang X., Huang H., Liu P. (2009). Berberine reduces fibronectin and collagen accumulation in rat glomerular mesangial cells cultured under high glucose condition. Mol. Cell. Biochem..

[29] Phang J.M., Pandhare J., Liu Y. (2008). The metabolism of proline as microenvironmental stress substrate. J. Nutr..

[30] Polyak K., Xia Y., Zweier J.L., Kinzler K.W., Vogelstein B. (1997). A model for p53-induced apoptosis. Nature.

[31] Maxwell S.A., Kochevar G.J. (2008). Identification of a p53-response element in the promoter of the proline oxidase gene. Biochem. Biophys. Res. Commun..

[32] Myara I., Myara A., Mangeot M., Fabre M., Charpentier C., Lemonnier A. (1984). Plasma prolidase activity: A possible index of collagen catabolism in chronic liver disease. Clin. Chem..

[33] Mock W.L., Green P.C., Boyer K.D. (1990). Specificity and pH dependence for acylproline cleavage by prolidase. J. Biol. Chem..

[34] Wang S.H., Zhi Q.W., Sun M.J. (2006). Dual activities of human prolidase. Toxicol. In Vitro.

[35] Adibi S.A., Mercer D.W. (1973). Protein digestion in human intestine as reflected in luminal, mucosal, and plasma amino acid concentrations after meals. J. Clin. Investig..

[36] Jackson S.H., Dennis A.W., Greenberg M. (1975). Iminodipeptiduria: A genetic defect in recycling collagen; a method for determining prolidase in erythrocytes. Can. Med. Assoc. J..

[37] Priest R.E., Davies L.M. (1969). Cellular proliferation and synthesis of collagen. Lab. Investig..

[38] Zhang M., White T.A., Schuermann J.P., Baban B.A., Becker D.F., Tanner J.J. (2004). Structures of the Escherichia coli PutA proline dehydrogenase domain in complex with competitive inhibitors. Biochemistry.

[39] Summitt C.B., Johnson L.C., Jonsson T.J., Parsonage D., Holmes R.P., Lowther W.T. (2015). Proline dehydrogenase 2 (PRODH2) is a hydroxyproline dehydrogenase (HYPDH) and molecular target for treating primary hyperoxaluria. Biochem. J..

[40] Hersey P., Watts R.N., Zhang X.D., Hackett J. (2009). Metabolic approaches to treatment of melanoma. Clin. Cancer Res..

[41] Warburg O. (1956). On respiratory impairment in cancer cells. Science.

[42] Ahn C.S., Metallo C.M. (2015). Mitochondria as biosynthetic factories for cancer proliferation. Cancer Metab..

[43] Hay N. (2016). Reprogramming glucose metabolism in cancer: Can it be exploited for cancer therapy?. Nat. Rev. Cancer.

[44] Frattaruolo L., Brindisi M., Curcio R., Marra F., Dolce V., Cappello A.R. (2020). Targeting the Mitochondrial Metabolic Network: A Promising Strategy in Cancer Treatment. Int. J. Mol. Sci..

[45] Filipp F.V., Ratnikov B., De Ingeniis J., Smith J.W., Osterman A.L., Scott D.A. (2012). Glutamine-fueled mitochondrial metabolism is decoupled from glycolysis in melanoma. Pigment. Cell Melanoma Res..

[46] Kowaloff E.M., Phang J.M., Granger A.S., Downing S.J. (1977). Regulation of proline oxidase activity by lactate. Proc. Natl. Acad. Sci. USA.

[47] Phang J.M., Liu W. (2012). Proline metabolism and cancer. Front. Biosci..

[48] Liu W., Glunde K., Bhujwalla Z.M., Raman V., Sharma A., Phang J.M. (2012). Proline oxidase promotes tumor cell survival in hypoxic tumor microenvironments. Cancer Res..

[49] Catchpole G., Platzer A., Weikert C., Kempkensteffen C., Johannsen M., Krause H., Jung K., Miller K., Willmitzer L., Selbig J. (2011). Metabolic profiling reveals key metabolic features of renal cell carcinoma. J. Cell. Mol. Med..

[50] Hirayama A., Kami K., Sugimoto M., Sugawara M., Toki N., Onozuka H., Kinoshita T., Saito N., Ochiai A., Tomita M. (2009). Quantitative metabolome profiling of colon and stomach cancer microenvironment by capillary electrophoresis time-of-flight mass spectrometry. Cancer Res..

[51] Ii M., Yamamoto H., Adachi Y., Maruyama Y., Shinomura Y. (2006). Role of matrix metalloproteinase-7 (matrylisin) in human cancer invasion, apoptosis, growth, and angiogenesis. Exp. Biol. Med..

[52] Laderoute K.R., Amin K., Calaoagan J.M., Knapp M., Le T., Orduna J., Foretz M., Viollet B. (2006). 5’-AMP-activated protein kinase (AMPK) is induced by low-oxygen and glucose deprivation conditions found in solid-tumor microenvironments. Mol. Cell. Biol..

[53] Wang W., Guan K.L. (2009). AMP-activated protein kinase and cancer. Acta Physiol..

[54] Hardie D.G. (2008). AMPK and Raptor: Matching cell growth to energy supply. Mol. Cell.

[55] Kato K., Ogura T., Kishimoto A., Minegishi Y., Nakajima N., Miyazaki M., Esumi H. (2002). Critical roles of AMP-activated protein kinase in constitutive tolerance of cancer cells to nutrient deprivation and tumor formation. Oncogene.

[56] D’Aniello C., Patriarca E.J., Phang J.M., Minchiotti G. (2020). Proline Metabolism in Tumor Growth and Metastatic Progression. Front. Oncol..

[57] Zareba I., Huynh T.Y.L., Kazberuk A., Teul J., Klupczynska A., Matysiak J., Surazynski A., Palka J. (2020). Overexpression of Prolidase Induces Autophagic Death in MCF-7 Breast Cancer Cells. Cell Physiol. Biochem..

[58] Zareba I., Surazynski A., Chrusciel M., Miltyk W., Doroszko M., Rahman N., Palka J. (2017). Functional Consequences of Intracellular Proline Levels Manipulation Affecting PRODH/POX-Dependent Pro-Apoptotic Pathways in a Novel in Vitro Cell Culture Model. Cell Physiol. Biochem..

[59] Zareba I., Celinska-Janowicz K., Surazynski A., Miltyk W., Palka J. (2018). Proline oxidase silencing induces proline-dependent pro-survival pathways in MCF-7 cells. Oncotarget.

[60] Huynh T.Y.L., Oscilowska I., Saiz J., Niziol M., Baszanowska W., Barbas C., Palka J. (2021). Metformin Treatment or PRODH/POX-Knock out Similarly Induces Apoptosis by Reprograming of Amino Acid Metabolism, TCA, Urea Cycle and Pentose Phosphate Pathway in MCF-7 Breast Cancer Cells. Biomolecules.

[61] Fearns C., Dowdle E.B. (1992). The desmoplastic response: Induction of collagen synthesis by melanoma cells in vitro. Int. J. Cancer.

[62] Gelse K., Pfander D., Obier S., Knaup K.X., Wiesener M., Hennig F.F., Swoboda B. (2008). Role of hypoxia-inducible factor 1 alpha in the integrity of articular cartilage in murine knee joints. Arthritis Res. Ther..

[63] Theron A.E., Nolte E.M., Lafanechère L., Joubert A.M. (2013). Molecular crosstalk between apoptosis and autophagy induced by a novel 2-methoxyestradiol analogue in cervical adenocarcinoma cells. Cancer Cell Int..

[64] Cechowska-Pasko M., Palka J., Wojtukiewicz M.Z. (2006). Enhanced prolidase activity and decreased collagen content in breast cancer tissue. Int. J. Exp. Pathol..

[65] Tang C.J., Xu J., Ye H.Y., Wang X.B. (2021). Metformin prevents PFKFB3-related aerobic glycolysis from enhancing collagen synthesis in lung fibroblasts by regulating AMPK/mTOR pathway. Exp. Ther. Med..

[66] Xu S., Yang Z., Jin P., Yang X., Li X., Wei X., Wang Y., Long S., Zhang T., Chen G. (2018). Metformin Suppresses Tumor Progression by Inactivating Stromal Fibroblasts in Ovarian Cancer. Mol. Cancer Ther..

[67] Karaszewski J., Zareba I., Guszczyn T., Darewicz B., Palka J. (2020). Verapamil and collagenase differentially affect collagen metabolism in experimental model of Peyronie’s disease. Mol. Cell. Probes.

[68] Baszanowska W., Misiura M., Oscilowska I., Palka J., Miltyk W. (2021). Extracellular Prolidase (PEPD) Induces Anabolic Processes through EGFR, beta1-integrin, and IGF-1R Signaling Pathways in an Experimental Model of Wounded Fibroblasts. Int. J. Mol. Sci..

[69] Misiura M., Guszczyn T., Oscilowska I., Baszanowska W., Palka J., Miltyk W. (2021). Platelet-Rich Plasma Promotes the Proliferation of Human Keratinocytes via a Progression of the Cell Cycle. A Role of Prolidase. Int. J. Mol. Sci..

[70] Natarajan S.K., Zhu W., Liang X., Zhang L., Demers A.J., Zimmerman M.C., Simpson M.A., Becker D.F. (2012). Proline dehydrogenase is essential for proline protection against hydrogen peroxide-induced cell death. Free. Radic. Biol. Med..

[71] Tallarita E., Pollegioni L., Servi S., Molla G. (2012). Expression in Escherichia coli of the catalytic domain of human proline oxidase. Protein Expr. Purif..

[72] Misiura M., Baszanowska W., Ościłowska I., Pałka J., Miltyk W. (2020). Prolidase Stimulates Proliferation and Migration through Activation of the PI3K/Akt/mTOR Signaling Pathway in Human Keratinocytes. Int. J. Mol. Sci..

[73] Lowry O.H., Rosebrough N.J., Farr A.L., Randall R.J. (1951). Protein measurement with the Folin phenol reagent. J. Biol. Chem..

[74] Klupczynska A., Misiura M., Miltyk W., Oscilowska I., Palka J., Kokot Z.J., Matysiak J. (2020). Development of an LC-MS Targeted Metabolomics Methodology to Study Proline Metabolism in Mammalian Cell Cultures. Molecules.

