# Supplementary material for: Proline Dehydrogenase/Proline Oxidase (PRODH/POX) Is Involved in the Mechanism of Metformin-Induced Apoptosis in C32 Melanoma Cell Line
Source: Int J Mol Sci. 2022 Feb 21;23(4):2354. doi: 10.3390/ijms23042354 (PMC8876342; doi:10.3390/ijms23042354)
Supplement: Supplementary file 1 [file ijms-23-02354-s001.zip › ijms-1592405-supplementary.pdf]

Supplementary Material

Article

PRODH/POX is involved in the mechanism of metformin-induced apoptosis in melanoma cells.

Ilona Oscilowska[1], Karol Rolkowski[2], Weronika Baszanowska[2], Thi Yen Ly Huynh[2], Sylwia Lewoniewska[2], Magdalena Nizioł[1], Magdalena Sawicka[1], Katarzyna Bielawska[1], Paweł Szoka[1], Wojciech Miltyk[1] and Jerzy Palka[2,*]

[1] Department of Analysis and Bioanalysis of Medicines, Medical University of Bialystok, Kilinskiego 1, 15-089 Bialystok, Poland, phone: +48-85-748-5845; e-mail: ilona.zareba@gmail.com (I.O.), katarzyna.bielawska@umb.edu.pl (K.B.), pawelszoka@wp.pl (P.S.), magdalena.niziol@umb.edu.pl (M.N.), sawicka.magdalenamaria@gmail.com (M.S.), wojciech.miltyk@umb.edu.pl (W.M.)

[2] Department of Medicinal Chemistry, Medical University of Bialystok, Kilinskiego 1, 15-089 Bialystok, Poland, phone: +48-85-748-5706; e-mail: karol.rolkowski@gmail.com (R.K.), w.baszanowska22@wp.pl (W.B.), htyly79@gmail.com (T.Y.L.H.), sylwialewoniewska123@wp.pl (S.L.), pal@umb.edu.pl (J.P.)

* Correspondence: pal@umb.edu.pl; Tel.: +48-85-748-5706

1. Results

1.1. *AMPK expression*

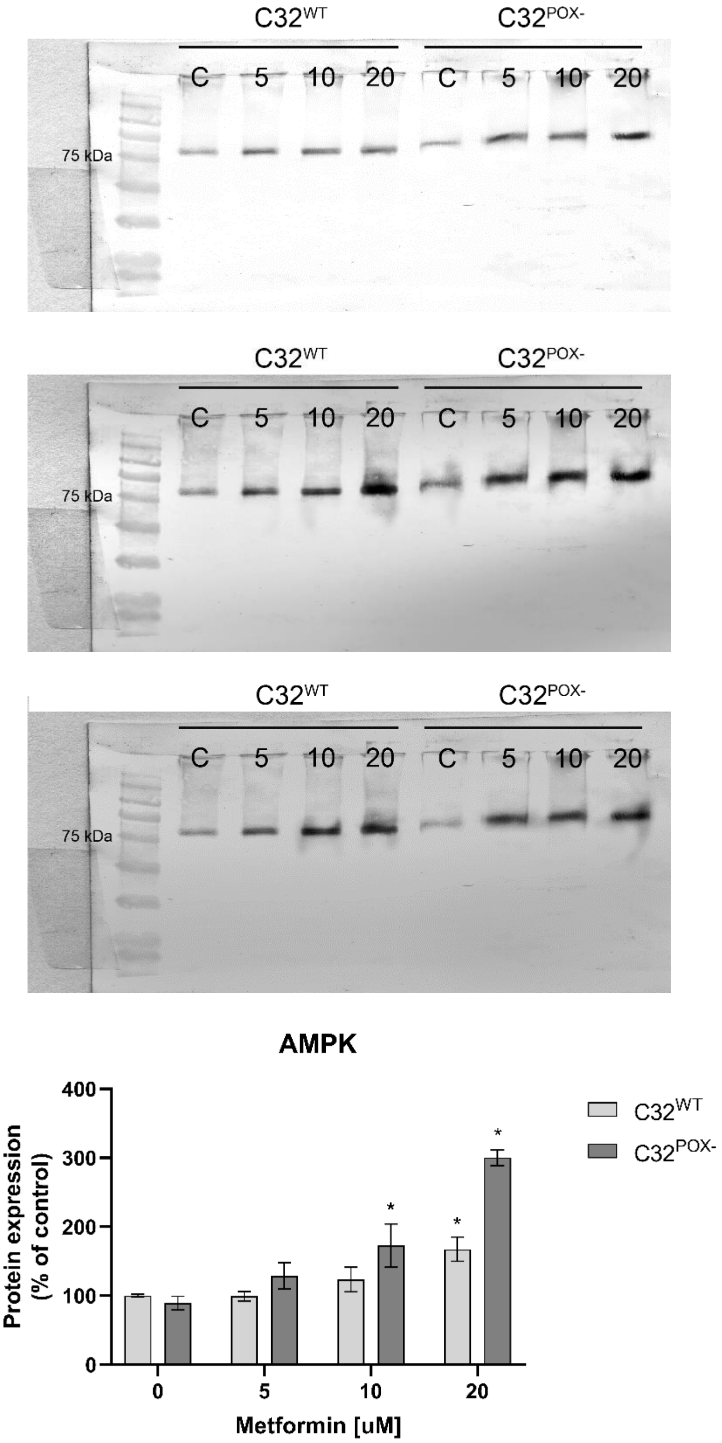

Figure S1 The AMPK expressions in C32^{WT} and C32^{POX-} cells cultured in DMEM. GAPDH expression was used as a loading control. The WB bands intensity of representative gels was quantified by densitometry and normalized to GAPDH. The densitometry values represent the mean (% of control) ± SD of three experiments, *P <0.001.

1.2. *PRODH/POX expression*

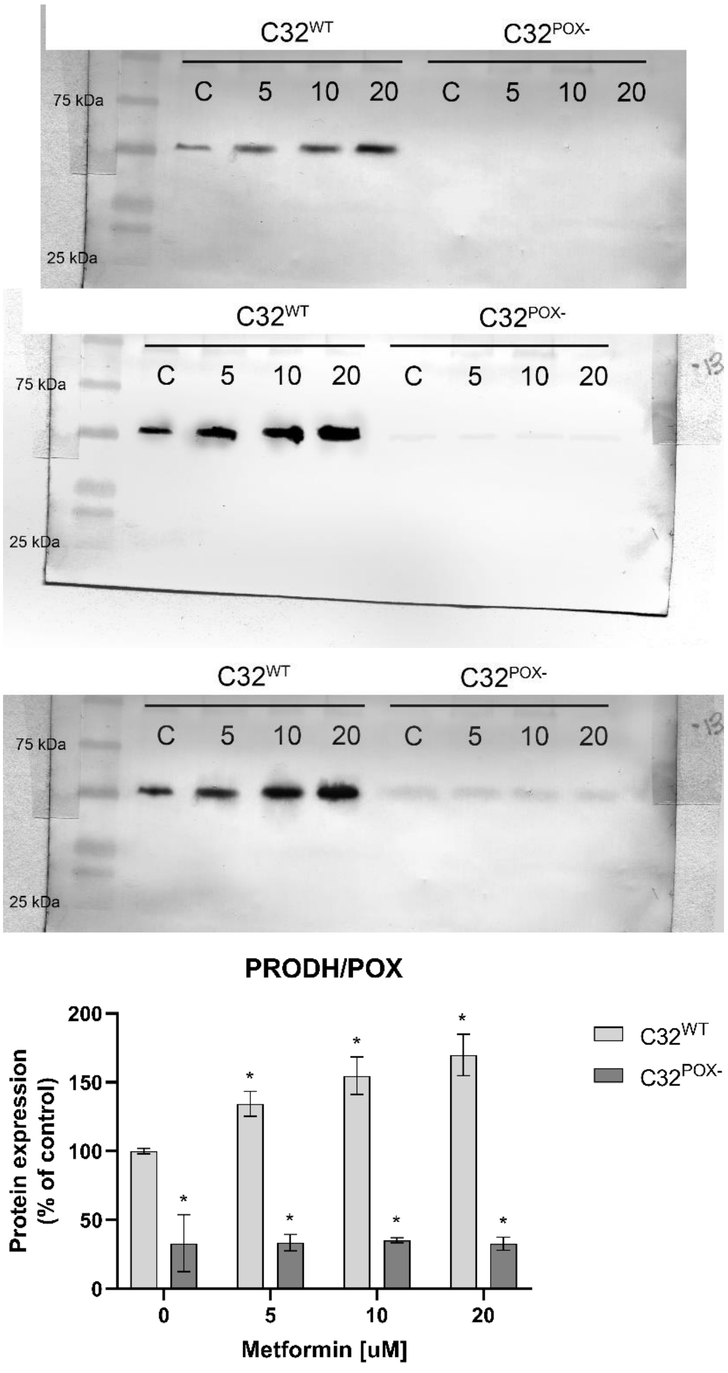

Figure S2 The PRODH/POX expressions in C32^{WT} and C32^{POX-} cells cultured in DMEM. GAPDH expression was used as a loading control. The WB bands intensity of representative gels was quantified by densitometry and normalized to GAPDH. The densitometry values represent the mean (% of control) ± SD of three experiments, *P <0.001.

1.3. Expression of Caspase-9 and cleaved-Caspase-9

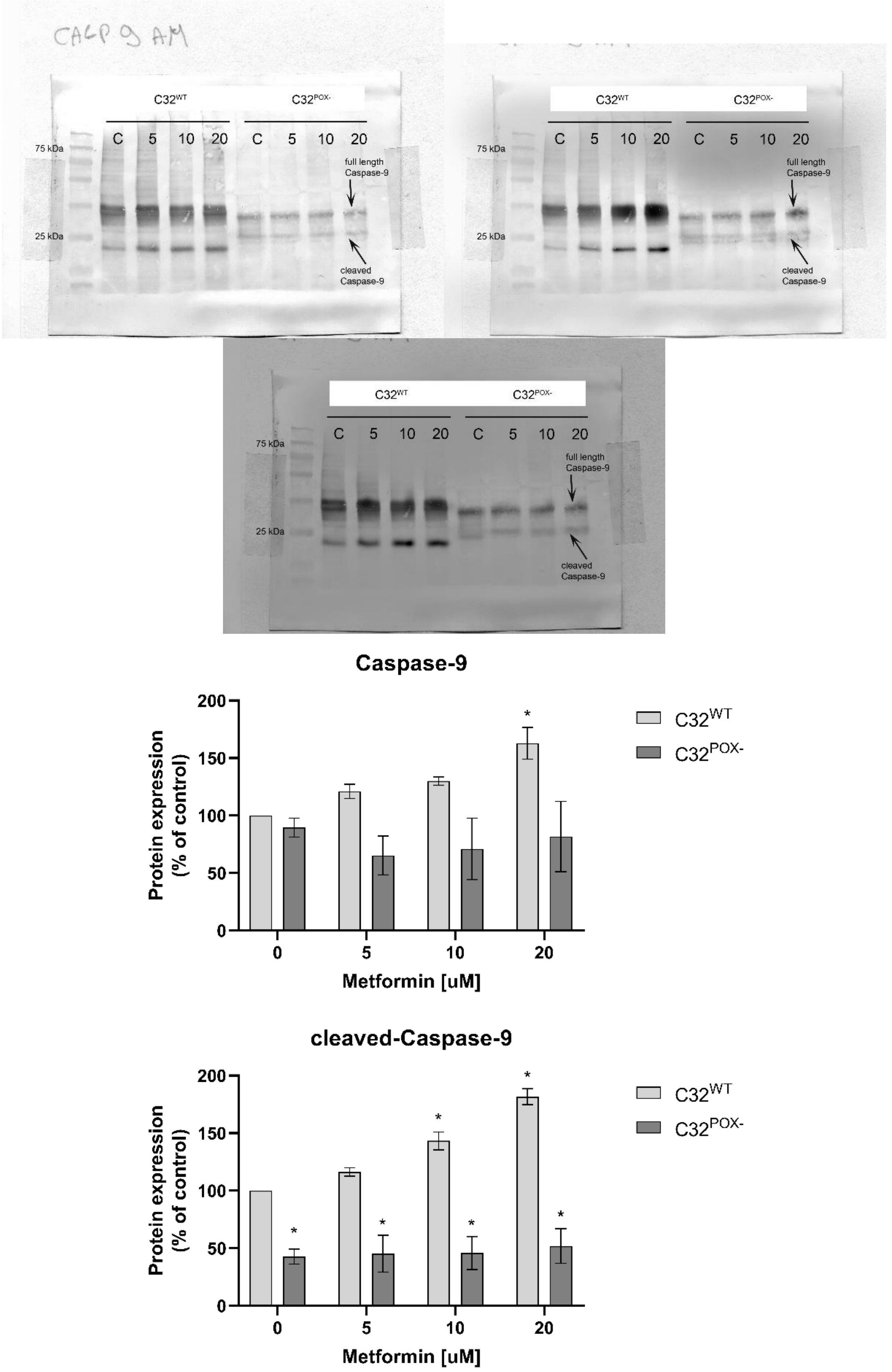

Figure S3 The expression of Caspase-9 and cleaved-Caspase-9 in C32^{WT} and C32^{POX-} cells cultured in DMEM. GAPDH expression was used as a loading control. The WB bands intensity of representative gels was quantified by densitometry and normalized to GAPDH. The densitometry values represent the mean (% of control) ± SD of three experiments, *P <0.001.

1.4. Caspase-3 expression

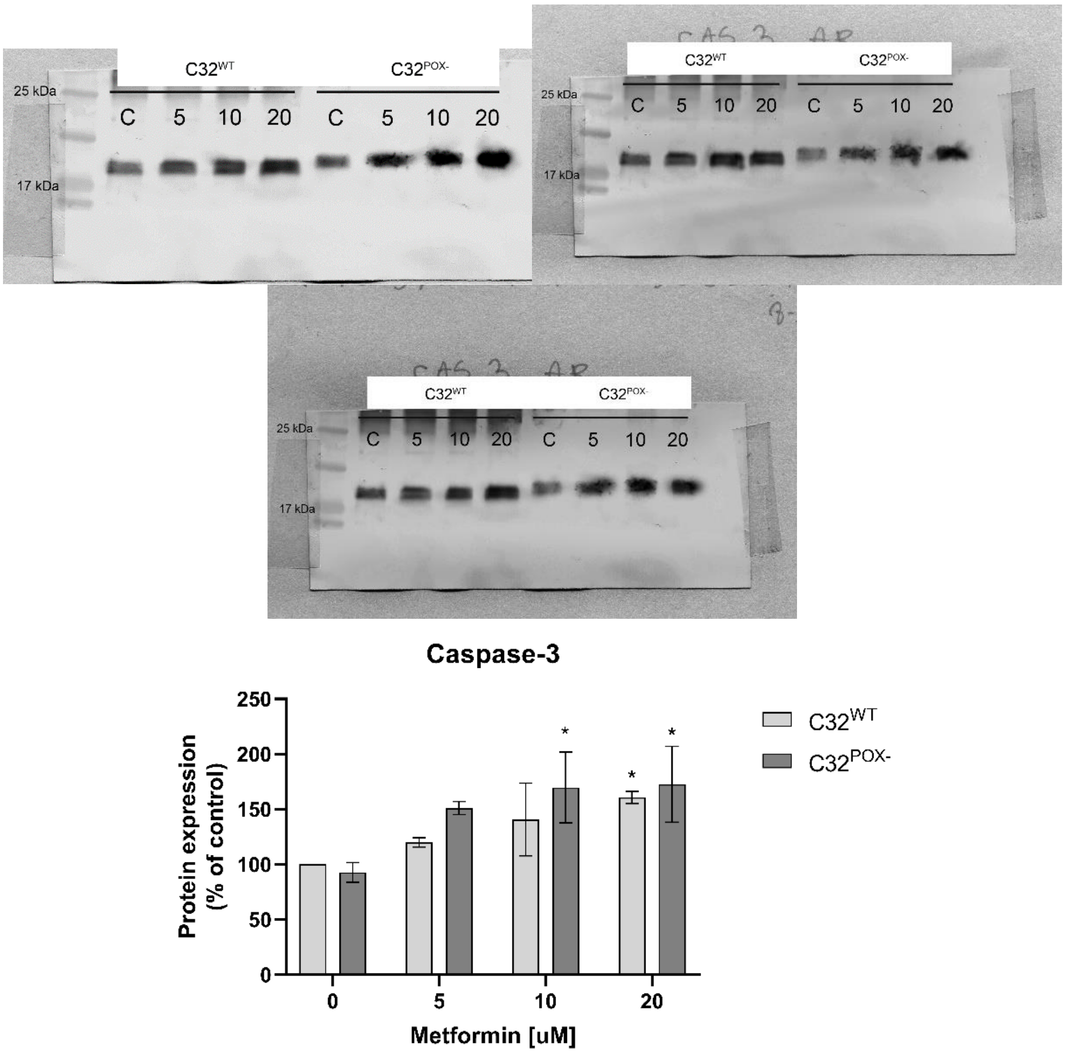

Figure S4 The Caspase-3 expressions in C32^{WT} and C32^{POX-} cells cultured in DMEM. GAPDH expression was used as a loading control. The WB bands intensity of representative gels was quantified by densitometry and normalized to GAPDH. The densitometry values represent the mean (% of control) ± SD of three experiments, *P <0.001.

1.5. Cleaved-Caspase-3 expression

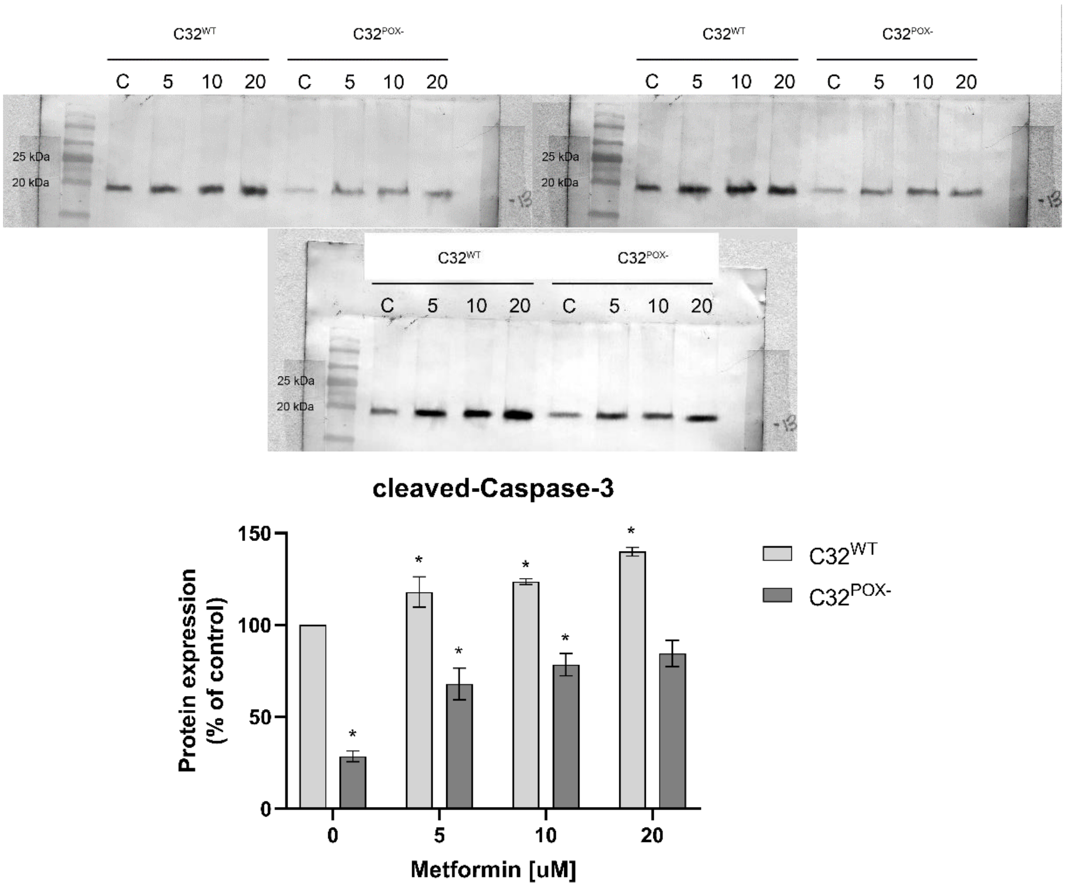

Figure S5 The cleaved-Caspase-3 expressions in C32^{WT} and C32^{POX-} cells cultured in DMEM. GAPDH expression was used as a loading control. The WB bands intensity of representative gels was quantified by densitometry and normalized to GAPDH. The densitometry values represent the mean (% of control) ± SD of three experiments, *P <0.001.

1.6. Expression of PARP and cleaved-PARP

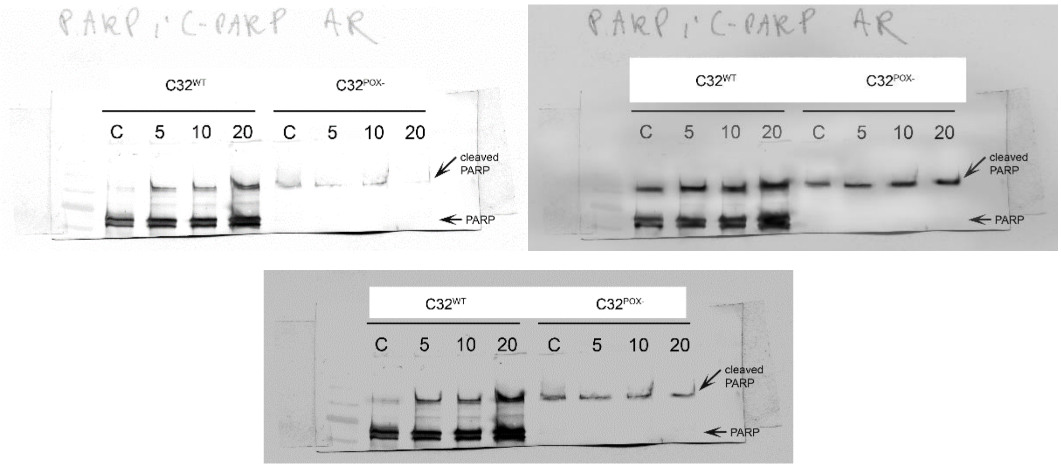

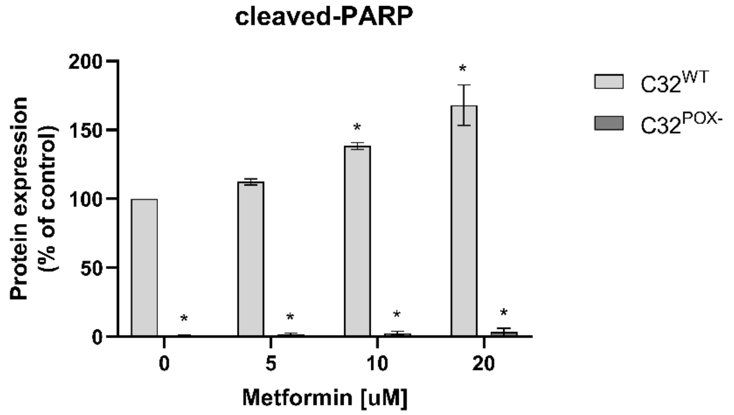

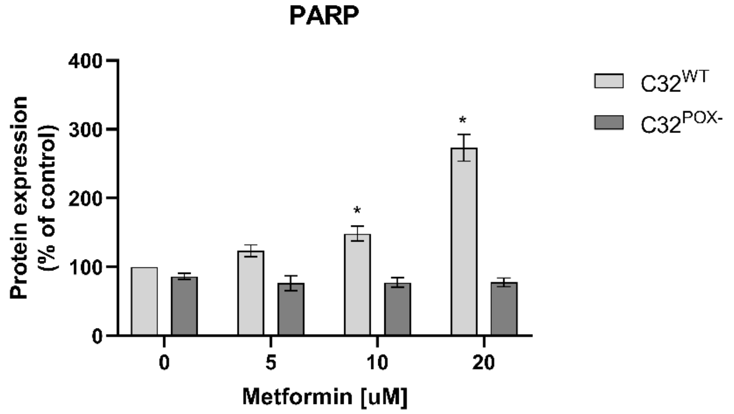

Figure S6 The expressions of PARP and cleaved-PARP in C32^{WT} and C32^{POX-} cells cultured in DMEM. GAPDH expression was used as a loading control. The WB bands intensity of representative gels was quantified by densitometry and normalized to GAPDH. The densitometry values represent the mean (% of control) ± SD of three experiments, *P <0.001.

1.7. Beclin-1 expression

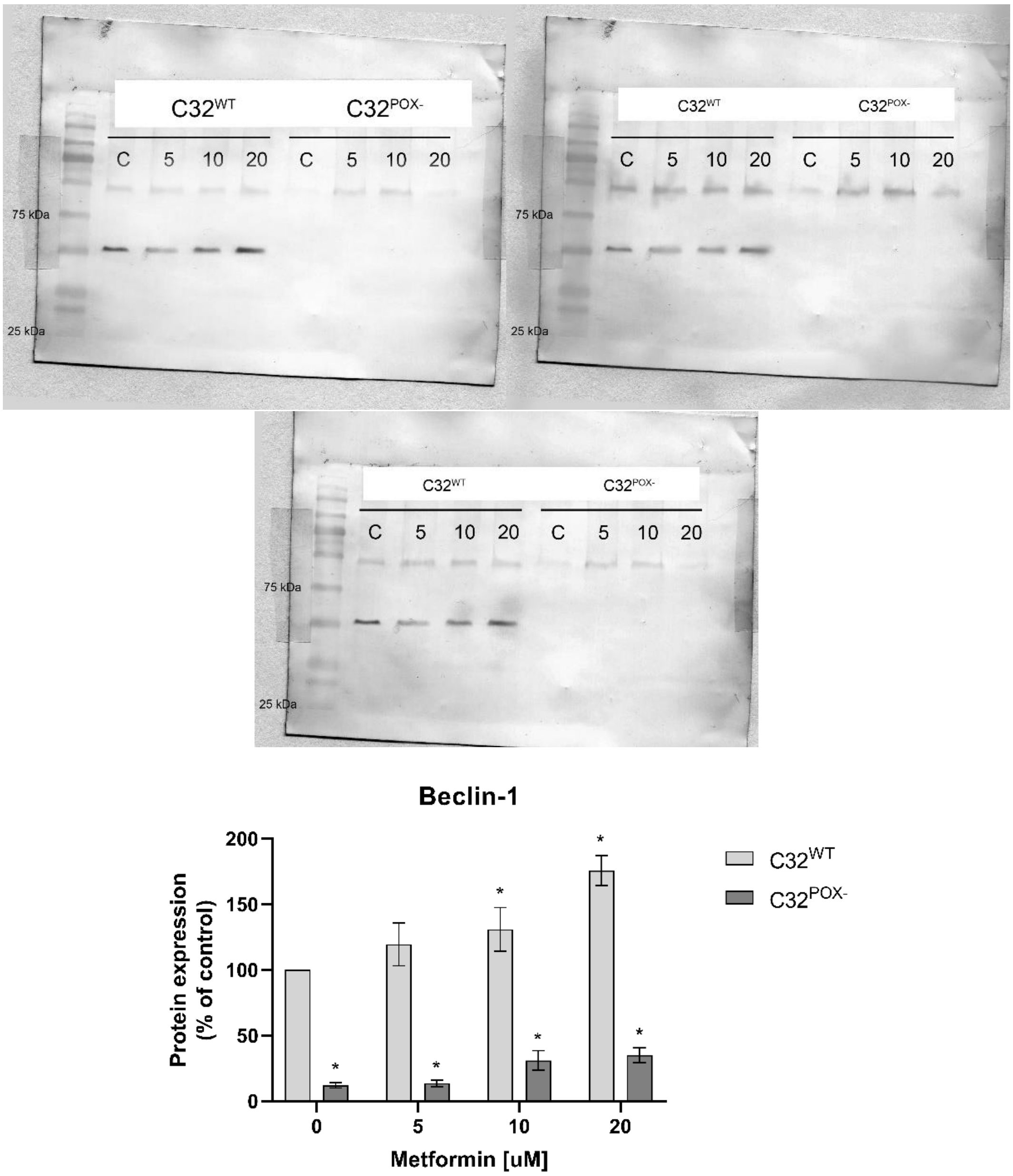

Figure S7 The Beclin-1 expressions in C32^{WT} and C32^{POX-} cells cultured in DMEM. GAPDH expression was used as a loading control. The WB bands intensity of representative gels was quantified by densitometry and normalized to GAPDH. The densitometry values represent the mean (% of control) ± SD of three experiments, *P <0.001.

1.8. *Atg7 expression*

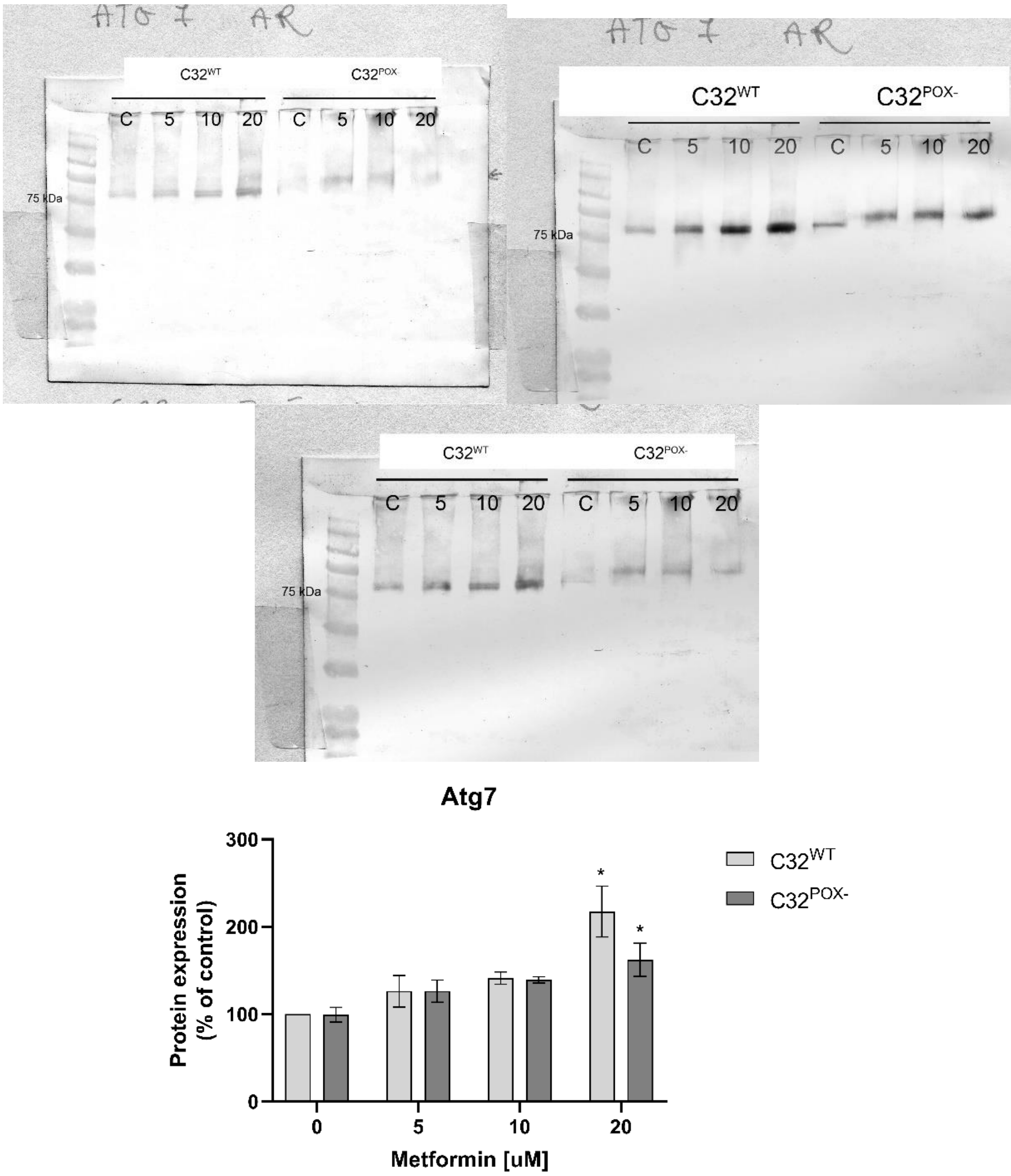

Figure S8 The Atg7 expressions in C32^{WT} and C32^{POX-} cells cultured in DMEM. GAPDH expression was used as a loading control. The WB bands intensity of representative gels was quantified by densitometry and normalized to GAPDH. The densitometry values represent the mean (% of control) ± SD of three experiments, *P <0.001.
